# Predicting Successful Weaning through Sonographic Measurement of the Rapid Shallow Breathing Index

**DOI:** 10.3390/jcm13164809

**Published:** 2024-08-15

**Authors:** Eunki Chung, Ah Young Leem, Su Hwan Lee, Young Ae Kang, Young Sam Kim, Kyung Soo Chung

**Affiliations:** 1 Division of Pulmonology, Department of Internal Medicine, National Health Insurance Service Ilsan Hospital, Goyang 10444, Republic of Korea; 2 Department of Internal Medicine, Yonsei University Graduate School of Medicine, Seoul 03722, Republic of Korea; 3 Division of Pulmonary and Critical Care Medicine, Department of Internal Medicine, Severance Hospital, Yonsei University College of Medicine, Seoul 03722, Republic of Korea

**Keywords:** diaphragm, mechanical ventilation, ultrasonography, ventilator weaning

## Abstract

**Background:** Diaphragmatic dysfunction correlates with weaning failure, highlighting the need to independently assess the diaphragm’s effects on weaning. We modified the rapid shallow breathing index (RSBI), a predictor of successful weaning, by incorporating temporal variables into existing ultrasound-derived diaphragm index to create a simpler index closer to tidal volume. **Methods:** We conducted a prospective observational study of patients who underwent a spontaneous breathing trial in the medical intensive care unit (ICU) at Severance Hospital between October 2022 and June 2023. Diaphragmatic displacement (DD) and diaphragm inspiratory time (Ti) were measured using lung ultrasonography. The modified RSBI was defined as follows: respiratory rate (RR) divided by DD was defined as D-RSBI, and RR divided by the sum of the products of DD and Ti on both sides was defined as DTi-RSBI. **Results:** Among the sonographic indices, DTi-RSBI had the highest area under the receiver operating characteristic (ROC) curve of 0.774 in ROC analysis, and a correlation was found between increased DTi-RSBI and unsuccessful extubation in a multivariable logistic regression analysis (adjusted odds ratio 0.02, 95% confidence interval 0.00–0.97). **Conclusions:** The DTi-RSBI is beneficial in predicting successful weaning in medical ICU patients.

## 1. Introduction

The importance of lung ultrasound in critical care medicine is steadily increasing [1,2]. Ultrasound has the advantage of being performed noninvasively and in real-time at the bedside, making it highly suitable for critically ill patients who require point-of-care monitoring [2]. Lung ultrasound is widely used in critically ill patients for diagnosis, monitoring treatment progress, and predicting prognosis [3,4,5]. Its utility is particularly emphasized in predicting weaning [6], which is an essential task in the management of mechanically ventilated patients.

Weaning factors that can be assessed through lung ultrasound include lung aeration, pleural effusion, and diaphragmatic function [6,7]. To evaluate the impact of lung aeration on weaning, the lung ultrasound score proposed in Bouhemad’s study is widely used [8,9,10], while for pleural effusion, the maximal interpleural distance or Balik’s formula is commonly utilized [11,12]. In assessing diaphragmatic function, previous studies have proposed ultrasound indicators such as diaphragmatic displacement (DD) and diaphragm thickening fraction (DTF) [13,14]. However, there are conflicting research results, indicating that these indicators may have limited predictive value for weaning [15], highlighting the need for the development of better indices to assess diaphragmatic function. To overcome these issues, previous studies have attempted to enhance predictive power by combining diaphragmatic ultrasound indicators with other metrics. One such approach involves combining it with the rapid shallow breathing index (RSBI), which is commonly used for weaning prediction [16].

The rapid shallow breathing index (RSBI) is defined as the ratio of respiratory rate (RR) to tidal volume (TV) [17]. The TV used in calculating the RSBI includes the TV formed by the diaphragm and that generated by the accessory inspiratory muscles [16]. Because diaphragmatic dysfunction is strongly associated with weaning failure [18], it is necessary to assess the independent impact of the diaphragm on weaning. Previous studies have investigated the replacement of TV with diaphragm indicators, such as DD [16] and DTF [14], measured using lung ultrasound in calculating RSBI. The modified RSBI proposed in previous research has shown an improvement in predicting weaning outcomes compared with conventional RSBI. This underscores the importance of evaluating the independent effects of the diaphragm. However, DD is an indicator that cannot exclude the influence of BMI [19], and DTF is susceptible to variations based on the measurement site [20]. Therefore, there is a need for the development of a diaphragm index that is more independent of covariate effects while still being easily measurable through lung ultrasound.

Thus, this study aimed to calculate a modified RSBI that incorporates temporal variables into existing ultrasound-derived diaphragm indices to obtain a simpler index closer to the TV. Subsequently, we assessed its utility compared to other lung ultrasound indices and the RSBI.

## 2. Materials and Methods

### 2.1. Study Design

We conducted a prospective observational study between October 2022 and June 2023 in the medical ICU of Severance Hospital, a tertiary referral hospital in the Republic of Korea. We included patients who met the following inclusion criteria for performing a spontaneous breathing trial (SBT): (1) fraction of inspired oxygen (FiO_2_) < 0.4 and < 8 cm H_2_O pressure support ventilation [21], (2) RR < 30/min, (3) hemodynamic stability status without or with low-dose vasopressors (dopamine or dobutamine < 5 μg/kg/min or norepinephrine < 0.05 μg/kg/min) [14], (4) improvement or resolution of the medical reason for intubation upon admission, (5) confirmed consciousness, and (6) no diaphragm paralysis. The eligibility for performing SBT was determined by the attending ICU physician, and the investigator did not intervene in clinical decisions. Mechanical ventilation was discontinued at the start of SBT, and simultaneously, the patient underwent spontaneous breathing using the same FiO_2_ value as used in mechanical ventilation via a T-tube circuit. During SBT, if the patient maintained RR < 30 breaths/min and SpO_2_ ≥ 95% without hemodynamic instability for 30 min, arterial blood gas analysis was conducted after its completion to confirm whether the patient met the extubation criteria. Lung ultrasonography was performed on participants whose immediate family provided consent for the study, and those who met the inclusion criteria. After the lung ultrasound was performed, the following exclusion criteria were applied: (1) time interval between the lung ultrasound and the extubation time ≥ 48 h, (2) time interval between the lung ultrasound and the first SBT ≥ 24 h, (3) patients who have neurological or mental problems that may affect extubation, and (4) the participants’ immediate family members did not consent to participate in the research. Two participants were excluded in the first criterion, and two more participants were excluded in the third criterion. The primary outcome was weaning success. The secondary outcomes were a hospital length of stay ≥ 14 days after ICU discharge and mortality after ICU discharge.

### 2.2. Procedure of Lung Ultrasound and Measurement of Lung Ultrasound Indices

A handheld ultrasound probe (Lumify S4-1, phased-array transducers 4–1 MHz; Philips, Amsterdam, The Netherlands) connected to a tablet computer (Galaxy A5; Samsung, Seoul, Republic of Korea) was used. The patient’s position during ultrasonography was supine, with elevation of the upper body at 30°.

Lung ultrasound indices related to factors that can influence weaning, such as lung aeration, pleural effusion, and diaphragmatic function, were individually measured. Lung aeration was assessed using the lung score, and we conducted a modified 12-point lung ultrasound examination based on the 6-point lung ultrasound examination, known as the blue point [22]. Horizontal lines were drawn 1 cm below the sternal angle and nipple levels, and measurements were taken at six points where these lines intersected with the midclavicular, midaxillary, and posterior axillary lines on the left and right sides. The lung scores were graded from 0 to 3 at each measurement point: 0 points for visible horizontal A-lines or ≤2 B-lines, 1 point for multiple B-lines, 2 points for multiple coalescent B-lines, and 3 points for lung consolidation [23].

Pleural effusion was indirectly assessed through ultrasound by measuring the pleural effusion volume. Pleural fluid volume was measured at a point resembling the posterolateral alveolar and pleural syndrome point, where the horizontal line at the nipple level intersects the posterior axillary line [22]. The total pleural fluid quantity was calculated for the right and left sides using Balik et al.’s formula, and the results were summed as follows: pleural volume (mL) = 20 × maximal distance between the parietal and visceral pleura during end-expiration (mm) [12].

**Figure 1:**
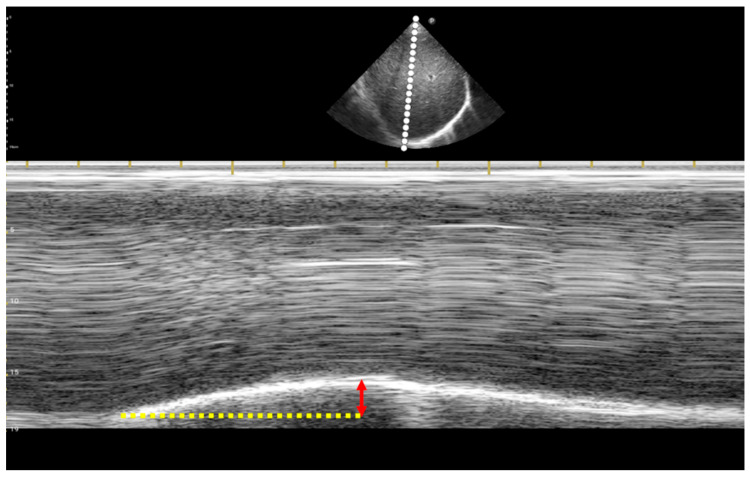
M-mode ultrasound of the diaphragm was used to measure diaphragmatic displacement (DD) and diaphragm inspiratory time (Ti). DD, which corresponds to the vertical distance from the end of the expiratory baseline to the peak inspiratory amplitude, is indicated by the red double-headed arrow, whereas Ti is represented by the yellow dashed line.

Diaphragmatic function was evaluated using two modified RSBI measurements: one was the RR divided by DD (D-RSBI) mentioned in prior research [16], and the other was the RR divided by the sum of the products of DD and diaphragm inspiratory time (Ti) on both sides (DTi-RSBI), which incorporates a temporal component and was proposed in this study. To calculate the D-RSBI, the DD measurement was required, while the DTi-RSBI necessitated measurements of both DD and Ti. DD and Ti were measured at the posterior third of both hemidiaphragm points when the maximum DD value was achieved during the three measurements. As shown in Figure 1, DD is the distance from the baseline, where diaphragmatic contraction begins in M-mode diaphragm ultrasound, to the maximum inspiratory amplitude, whereas Ti is the time taken to reach this point. The RR was recorded based on the fastest rate observed during measurements. The formula is as follows: D-RSBI = RR ÷ [0.5 × (right DD + left DD)]; the sum of the products of DD and Ti on both sides (DTi) = (right DD × right Ti) + (left DD × left Ti); DTi-RSBI = RR ÷ DTi.

Cardiac function is also known to affect weaning [24], and in this study, we assessed ejection fraction (EF) alongside lung ultrasonography. The EF was estimated by measuring the left ventricle end-diastolic and end-systolic diameters using the M-mode in the parasternal short-axis view and applying the Teichholz formula [25].

### 2.3. Statistical Analysis

Pearson’s chi-squared test was used to compare categorical variables. For continuous variables, normality was tested using the Shapiro–Wilk test, and the Kruskal–Wallis test was used for non-normal distributions. The area under the receiver operating characteristic (ROC) curve and the cutoff value for the maximum Youden index were calculated using the ROC curve for each lung ultrasound index and RSBI. The sample size was calculated based on the following values. With a weaning failure rate of 29% mentioned in previous literature [26], an area under the receiver operating characteristic curve (AUC) value of more than 0.75 was considered acceptable for diagnostic accuracy. Using a Type I error and Type II error of 0.1 (90% power), the sample size was calculated to be 30 participants. Subsequently, considering an anticipated dropout rate of 10%, the final sample size was determined to be 34 participants. Covariates were established based on correlation analysis between clinical variables and the modified RSBI. Using the covariates (age, sex, and body mass index [BMI]) as references, multivariable analyses using a logistic regression model were performed to compare the predicted values for each modified RSBI. In the overall analysis, *p*-values < 0.05 were considered statistically significant. R software (v.4.2.1; R Foundation for Statistical Computing, Vienna, Austria) was used for the statistical analysis.

### 2.4. Data Collection, Extubation Criteria, and Weaning Success Definition

Demographic characteristics, the Charlson Comorbidity Index (CCI), and reasons for ICU admission were collected at the time of ICU admission. The ejection fraction (EF) and lung ultrasound indices were measured on the day of lung ultrasonography. Sequential organ failure assessment (SOFA) scores were calculated at the time of ICU admission and extubation. The extubation criteria used in this study were as follows: (1) pH of 7.35–7.45, pCO_2_ < 45 mmHg, and pO_2_ > 60 mmHg on arterial blood gas analysis (ABGA) performed within 30 min after SBT, (2) RR < 30 breaths/min during SBT, (3) hemodynamically stable during SBT, and (4) responsive to verbal commands. If the first SBT satisfied all the extubation criteria, the attending ICU physician determined that the patient was ready for weaning and informed the investigator. Weaning success was defined as successful extubation without tracheostomy regardless of reintubation, and the patient was placed on nasal O_2_ or room air at the time of ICU discharge. In our study, patients classified as having weaning failure were those who died within two weeks after initiation of mechanical ventilation without meeting the extubation criteria, or those who underwent tracheostomy after more than two weeks of intubation.

## 3. Results

Of the 39 patients who met the inclusion criteria and were enrolled, 2 were excluded due to an interval of ≥48 h between lung ultrasonography and extubation, and 2 patients who underwent tracheostomy without attempting extubation were excluded. Subsequently, 35 patients were included in the analysis.

### 3.1. Baseline Characteristics

Table 1 compares the baseline characteristics of patients with weaning success and those with weaning failure. Of the 35 patients, 74.3% were male, and the median age was 67.0 (59.0–75.0) years. The median duration of mechanical ventilation was 10.0 days. The weaning success group was older (71.0 years vs. 60.0 years) and had a shorter ICU duration than in the weaning failure group. In the weaning failure group, tracheostomy was performed in eight patients (88.9%), and re-intubation within 48 h was necessary for six patients (66.7%). However, no statistically significant differences between the groups were observed in the other demographic characteristics, CCI, reasons for ICU admission, SOFA score at ICU admission and extubation, EF, and duration of mechanical ventilation.

### 3.2. ROC Analysis for Lung Ultrasonography Indices and Modified RSBI

**Figure 2:**
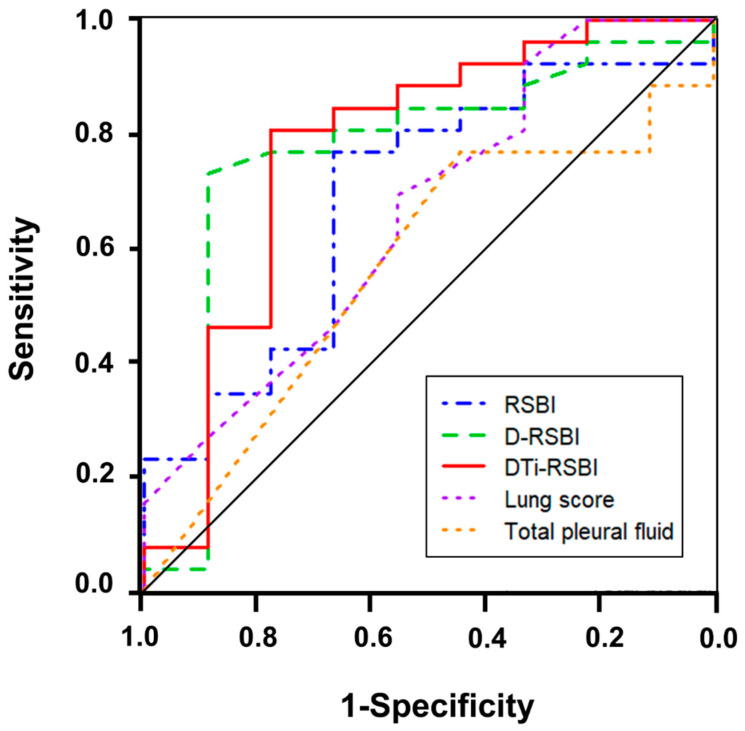
ROC curves for lung ultrasound indices and the rapid shallow breathing index (RSBI). D-RSBI, respiratory rate divided by half the sum of the right and left diaphragmatic displacements; DTi-RSBI, respiratory rate divided by the product of diaphragmatic displacement and diaphragm inspiratory time.

As shown in Figure 2, the DTi-RSBI exhibited the highest AUC value (0.774) among the sonographic indices. The D-RSBI had a similar AUC value of 0.765, whereas the RSBI, lung score, and total pleural fluid had relatively lower AUC values of 0.688, 0.660, and 0.568, respectively. The cutoff value of the DTi-RSBI was 1.13, with 80.8% sensitivity and 77.8% specificity (Supplementary Table S1).

### 3.3. Correlation Analysis between Clinical Variables and Modified RSBI

To determine the covariates to be included in the multivariable analysis, we investigated the correlations between the clinical variables and the D-RSBI and DTi-RSBI, which are modified RSBI (Supplementary Table S2). Statistically significant negative correlations were observed only between D-RSBI and BMI, and between DTi-RSBI and BMI. Therefore, in the multivariable analysis, we included BMI as a covariate along with demographic variables (age and sex).

### 3.4. Univariable and Multivariable Analysis for Modified RSBI

The logistic regression results for weaning success are presented in Table 2. D-RSBI (odds ratio [OR] 0.15, 95% confidence interval [CI] and DTi-RSBI (OR 0.07, 95% CI 0.01–0.67) were statistically significant in the univariable analysis. In the subsequent multivariable analysis, D-RSBI produced non-significant results in model 1 (adjusted OR [aOR] 0.14, 95% CI 0.02–1.05) and model 3 (aOR 0.11, 95% CI 0.01–1.67). However, model 2 (aOR 0.06, 95% CI 0.00–0.72) and model 4 (aOR 0.02, 95% CI 0.00–0.97) yielded significant results for DTi-RSBI, indicating a significant association between higher DTi-RSBI values and unsuccessful extubation. Additionally, a multivariable analysis was conducted for secondary outcomes, with the results presented in Supplementary Tables S3 and S4. An increase in DTi-RSBI was found to be associated with a hospital length of stay ≥ 14 days after ICU discharge, whereas no such association was observed for D-RSBI. However, neither D-RSBI nor DTi-RSBI demonstrated an association with mortality after ICU discharge.

## 4. Discussion

This study aimed to enhance the weaning indicator by incorporating DTi measured using pulmonary ultrasonography into the RSBI. The research findings indicated that the DTi-RSBI demonstrated superior predictive accuracy for weaning success in the multivariable analysis compared to the conventional and modified RSBI utilized in previous studies.

Spadaro et al. demonstrated that replacing tidal volume with DD to calculate D-RSBI can enhance the predictive ability for weaning compared with the conventional RSBI [16]. Likewise, in a study conducted by Song et al., the utilization of diaphragmatic excursion yielded similar outcomes [14]. However, even if they have similar DD, when the inspiratory time lengthens the decreased inspiration flow reduces the work of breathing, which can affect weaning success [27,28]. Because of this, Palkar et al. indirectly calculated the work performed by the diaphragm using the product of diaphragmatic excursion and inspiratory time, termed the excursion-time (E-T) index. They demonstrated that an increased E-T index is associated with successful weaning [28]. Therefore, we expected that additional consideration of inspiration time in D-RSBI would improve the predictive power of weaning by more accurately reflecting the work performed through the diaphragm. Our research findings showed that the AUC value was highest in DTi-RSBI, and the multivariable logistic regression results indicated that high DTi-RSBI was associated with weaning failure, demonstrating the utility of DTi-RSBI. Additionally, DTi-RSBI was found to be associated with the secondary outcome of hospital length of stay ≥ 14 days after ICU discharge in multivariable analysis, whereas no such association was observed with D-RSBI. Therefore, it is possible to confirm that DTi-RSBI could also be usefully utilized in predicting outcomes after ICU discharge.

In our study, the lung score and total pleural fluid, concurrently investigated with DTi, were known from previous research to help predict weaning success [6]. However, because this study focused on internal medicine patients with numerous comorbidities, underlying chronic lung diseases such as interstitial lung disease or chronic obstructive pulmonary disease may have affected the lung score. Furthermore, in patients with conditions such as heart or renal failure, chronic pleural effusion may be present, contributing to a lower predictive value for weaning success than DTi-RSBI.

The reported rate of unsuccessful weaning after a single SBT ranges from 26 to 42% [29]. Our study observed a weaning failure rate of 25.7%, comparable to that reported in other studies. Our study’s RSBI and D-RSBI cutoff values were 47.3 and 1.15, respectively. Spadaro et al. reported RSBI and D-RSBI cutoff values of 62 and 1.3, respectively, whereas Abbas et al. reported cutoff values of 70 and 1.9 [16,30]. Considering this discrepancy, it cannot be ruled out that our study participants may have included a relatively high proportion of patients for whom weaning was challenging.

The DTi-RSBI identified in this study is a relatively simple index to obtain, but it has the drawback of requiring lung ultrasound for clinical application. To apply the DTi-RSBI more cost- and time-effectively in clinical settings, one approach could be to use RSBI, which does not require lung ultrasound for initial screening. Subsequently, DTi-RSBI could be conducted to identify patients suitable for weaning attempts. This approach would reduce the number of patients needing lung ultrasound, allowing the researchers to focus on those with a higher likelihood of successful weaning, thus making the application of DTi-RSBI in clinical practice more efficient.

The advantages of this study are as follows: first, all lung ultrasonography examinations were conducted by a single examiner, eliminating variability among measurers. Second, we estimated weaning failure using the DD, which may not have been significantly influenced by the measurer’s skill level. There was little difference in DD between the mid and posterior portions of the diaphragm, indicating ease of measurement compared with DTF, which can be influenced by the measurement location [20,31]. Additionally, we enhanced the predictability of weaning failure by simultaneously measuring simple and easily obtainable Ti and DD.

However, this study had some limitations. First, since this study was conducted at a single center and by a single ultrasound examiner, it may not account for national and regional variations and may not be generalizable to multicenter studies that involve multiple examiners. Therefore, future studies with larger and more diverse participants across multiple centers are necessary. However, ultrasound measurements by the same examiner in the same environment can be considered as an advantage of environmental homogeneity. Second, this study was conducted with medical ICU patients, which limits the direct applicability of the findings to surgical ICU patients who receive postoperative care following elective surgeries. Patients undergoing elective surgery often have mild or no significant medical issues that could complicate the surgery, suggesting that weaning might be easier. However, surgery itself could function as a risk factor, potentially impacting weaning by causing issues such as type 3 respiratory failure. Therefore, including surgical ICU patients in future multicenter studies could address these issues. Third, inherent ultrasound characteristics, along with patient factors, may impact the reliability of measurements. Since ultrasound measurements are conducted in real-time, variations in the patient’s level of sedation or the occurrence of breakthrough pain at the time of measurement could result in index values that deviate from the daily average. Finally, this study did not evaluate the role of inflammatory mediators in weaning. Previous research has shown that elevated levels of inflammatory mediators, such as interleukin-6, -8, -10, -18, and tumor necrosis factor alpha, are associated with weaning failure [32,33,34]. However, since inflammatory mediators were not measured in this study, their potential impact on weaning could not be ruled out. Future research that includes the measurement of these mediators could provide a clearer assessment of the effect of DTi-RSBI on weaning.

## 5. Conclusions

DTi-RSBI, which incorporates DTi measured using lung ultrasound into the traditional RSBI, is beneficial in predicting successful weaning in medical ICU patients. Considering that this study’s findings may contribute to determining weaning timing in medical ICU patients, further validation of the clinical efficacy of DTi-RSBI is essential through subsequent large-scale studies.

## Figures and Tables

**Table 1:** Baseline characteristics of patients with weaning success and failure.

Characteristics	Total Participants (N = 35)	Weaning Failure (N = 9)	Weaning Success (N = 26)	*p*-Value
Age (year)	67.0 (59.0–75.0)	60.0 (57.0–66.0)	71.0 (61.8–77.8)	0.038
Male, no (%)	26 (74.3)	7 (77.8)	19 (73.1)	1.000
BMI (kg/m^2^)	23.8 (20.7–27.4)	21.0 (17.0–25.8)	24.3 (22.3–27.7)	0.101
Smoking status (current or former), no (%)	16 (45.7)	3 (33.3)	13 (50.0)	0.460
CCI	6.0 (5.0–8.0)	6.0 (3.5–6.5)	6.5 (5.0–8.3)	0.093
Reason for ICU admission, no (%)				1.000
Cardiovascular	6 (17.1)	1 (11.1)	5 (19.2)	
Respiratory	29 (82.9)	8 (88.9)	21 (80.8)	
SOFA at ICU admission	9.0 (7.0–11.0)	10.0 (8.5–10.5)	9.0 (6.8–11.0)	0.675
SOFA at extubation	5.0 (3.0–7.0)	6.0 (5.0–7.5)	4.0 (2.8–7.0)	0.093
EF < 50%, no (%)	12 (34.3)	4 (44.4)	8 (30.8)	0.456
Duration of mechanical ventilation (day)	10.0 (8.0–14.0)	12.0 (10.5–13.0)	9.0 (7.8–14.0)	0.382
ICU duration (day)	16.0 (10.0–24.0)	40.0 (23.5–65.0)	11.5 (9.0–18.0)	<0.001
Deceased patients, no (%)	8 (22.9)	4 (44.4)	4 (15.4)	0.162
Right DD (mm)	18.0 (12.0–22.0)	13.0 (10.5–16.5)	20.0 (12.8–23.5)	0.051
Left DD (mm)	16.0 (12.0–23.0)	13.0 (11.0–24.0)	17.5 (12.0–23.0)	0.382
Right Ti (sec)	0.6 (0.6–0.8)	0.6 (0.6–0.8)	0.7 (0.6–0.8)	0.781
Left Ti (sec)	0.6 (0.5–0.8)	0.6 (0.6–0.7)	0.7 (0.5–0.8)	0.781
DTi (mm×sec)	20.0 (15.5–30.8)	17.8 (13.5–25.8)	20.2 (17.0–31.9)	0.149
RSBI	38.5 (32.5–59.0)	58.8 (33.9–75.9)	35.2 (31.3–47.5)	0.101
D-RSBI	1.04 (0.83–1.46)	1.46 (1.23–1.97)	0.98 (0.79–1.20)	0.018
DTi-RSBI	0.93 (0.63–1.20)	1.29 (0.95–1.64)	0.81 (0.61–1.06)	0.015
Lung score	3.0 (2.0–5.0)	5.0 (2.0–9.0)	3.0 (1.0–5.0)	0.160
Total pleural fluid	0.0 (0.0–220.0)	0.0 (0.0–230.0)	0.0 (0.0–65.0)	0.565

BMI, body mass index; CCI, Charlson Comorbidity Index; ICU, intensive care unit; SOFA, sequential organ failure assessment; EF, ejection fraction; DD, diaphragmatic displacement; Ti, diaphragm inspiratory time; DTi, product of diaphragmatic displacement and diaphragm inspiratory time; RSBI, rapid shallow breathing index; D-RSBI, respiratory rate divided by half the sum of the right and left diaphragmatic displacement; DTi-RSBI, respiratory rate divided by the product of diaphragmatic displacement and diaphragm inspiratory time.

**Table 2:** Univariable and multivariable analyses of weaning success.

	Univariable Analysis		Multivariable Analysis (Model 1) ^a^		Multivariable Analysis (Model 2) ^a^		Multivariable Analysis (Model 3) ^b^		Multivariable Analysis (Model 4) ^b^	
	OR (95% CI)	*p*-Value	aOR (95% CI)	*p*-Value	aOR (95% CI)	*p*-Value	aOR (95% CI)	*p*-Value	aOR (95% CI)	*p*-Value
Age	1.06 (0.99–1.13)	0.120	1.08 (0.99–1.17)	0.078	1.08 (1.00–1.18)	0.062	1.05 (0.94–1.17)	0.406	1.06 (0.95–1.18)	0.287
Male	0.78 (0.13–4.67)	0.781	1.10 (0.14–8.84)	0.931	1.30 (0.15–11.47)	0.812	0.36 (0.01–10.82)	0.555	0.66 (0.02–24.79)	0.824
BMI (kg/m^2^)	1.13 (0.95–1.33)	0.164	1.06 (0.87–1.30)	0.556	1.04 (0.85–1.29)	0.692	1.05 (0.84–1.32)	0.665	1.02 (0.79–1.31)	0.909
Smoking	2.00 (0.41–9.76)	0.391					1.42 (0.12–16.4)	0.777	1.11 (0.07–18.13)	0.943
CCI	1.41 (0.97–2.03)	0.069					1.53 (0.90–2.61)	0.115	1.71 (1.00–2.93)	0.052
SOFA at extubation	0.75 (0.52–1.07)	0.110					0.73 (0.42–1.29)	0.279	0.80 (0.42–1.51)	0.484
EF < 50%	0.56 (0.12–2.63)	0.459					1.28 (0.06–25.72)	0.874	1.08 (0.04–28.16)	0.961
Duration of mechanical ventilation (day)	0.91 (0.73–1.14)	0.412					0.94 (0.66–1.34)	0.742	0.90 (0.61–1.33)	0.592
D-RSBI	0.15 (0.03–0.82)	0.029	0.14 (0.02–1.05)	0.055			0.11 (0.01–1.67)	0.112		
DTi-RSBI	0.07 (0.01–0.67)	0.022			0.06 (0.00–0.72)	0.027			0.02 (0.00–0.97)	0.048

^a^ Models 1 and 2 were adjusted for age, sex, and BMI. ^b^ Models 3 and 4 were adjusted for all variables. OR, odds ratio; aOR, adjusted odds ratio; CI, confidence interval; BMI, body mass index; CCI, Charlson Comorbidity Index; SOFA, sequential organ failure assessment; EF, ejection fraction; RSBI, rapid shallow breathing index; DTi, product of diaphragmatic displacement and diaphragm inspiratory time; D-RSBI, respiratory rate divided by half the sum of the right and left diaphragmatic displacements; DTi-RSBI, respiratory rate divided by the product of diaphragmatic displacement and diaphragm inspiratory time.

## Data Availability

The datasets used or analyzed during the current study are available from the corresponding author on reasonable request.

## Supplementary Materials

The following supporting information can be downloaded at: https://www.mdpi.com/article/10.3390/jcm13164809/s1, Table S1. The receiver operating characteristic curve showed the rapid shallow breathing index (RSBI) and weaning indices measured using lung ultrasound. Table S2. Correlation analysis between clinical variables and diaphragmatic indices measured using lung ultrasonography. Table S3. Univariable and multivariable analyses of hospital length of stay ≥ 14 days after ICU discharge. Table S4. Univariable and multivariable analyses of mortality after ICU discharge.

## Abbreviations

aOR	adjusted odd ratio
AUC	area under the receiver operating characteristic curve
BMI	body mass index
CCI	Charlson Comorbidity Index
CI	confidence interval
DD	diaphragmatic displacement
DTF	diaphragm thickening fraction
D-RSBI	respiratory rate divided by half the sum of the right and left diaphragmatic displacement
DTi-RSBI	respiratory rate divided by the product of diaphragmatic displacement and diaphragm inspiratory time
EF	ejection fraction
FiO_2_	fraction of inspired oxygen
ICU	intensive care unit
ROC	receiver operating characteristic
RR	respiratory rate
RSBI	rapid shallow breathing index
SBT	spontaneous breathing trial
SOFA	sequential organ failure assessment
Ti	diaphragm inspiratory time
TV	tidal volume
