# Supplementary material for: Predicting Successful Weaning through Sonographic Measurement of the Rapid Shallow Breathing Index
Source: J Clin Med. 2024 Aug 15;13(16):4809. doi: 10.3390/jcm13164809 (PMC11355280; doi:10.3390/jcm13164809)
Supplement: Supplementary file 1 [file jcm-13-04809-s001.zip › jcm-3112045-supplementary.pdf]

Predicting successful weaning through sonographic measurement of the rapid shallow breathing index

Eunki Chung[a, b], Ah Young Leem[c], Su Hwan Lee[c], Young Ae Kang[c], Young Sam Kim[c], Kyung Soo Chung[c]

[a]Division of Pulmonology, Department of Internal Medicine, National Health Insurance Service Ilsan Hospital, Goyang, Republic of Korea

[b]Yonsei University Graduate School of Medicine, Seoul, Republic of Korea

[c]Division of Pulmonary and Critical Care Medicine, Department of Internal Medicine, Severance Hospital, Yonsei University College of Medicine, Seoul, Republic of Korea

Supplementary information

Supplementary Table S1. The receiver operating characteristic curve values for the rapid shallow breathing index (RSBI) and weaning indices measured during lung ultrasonography

	AUC	Cutoff value	Sensitivity (%)	Specificity (%)
**RSBI**	0.688	47.30	76.9	66.7
**D-RSBI**	0.765	1.15	73.1	88.9
**DTi-RSBI**	0.774	1.13	80.8	77.8
**Lung score**	0.660	7.50	92.3	33.3
**Total pleural fluid**	0.568	100.00	76.9	44.4

AUC, area under the curve; RSBI, Rapid shallow breathing index; DD, diaphragmatic displacement; Ti, diaphragm inspiratory time; DTi, Product of diaphragmatic displacement and inspiratory time; D-RSBI, respiratory rate divided by half the sum of the right and left diaphragmatic displacements; DTi-RSBI, respiratory rate divided by the product of diaphragmatic displacement and inspiratory time.

Supplementary Table S2. Correlation analysis between clinical variables and diaphragmatic indices measured using lung ultrasonography

D-RSBI						
Age	1.00					
BMI	0.001	1.00				
CCI	0.322	-0.032	1.00			
SOFA	-0.078	-0.171	0.087	1.00		
Intubation period	-0.022	0.004	0.143	0.213	1.00	
D-RSBI	0.094	-0.408*	0.070	-0.012	0.121	1.00
	Age	BMI	CCI	SOFA	Intubation period	D-RSBI

DTi-RSBI						
Age	1.00					
BMI	0.001	1.00				
CCI	0.322	-0.032	1.00			
SOFA	-0.078	-0.171	0.087	1.00		
Intubation period	-0.022	0.004	0.143	0.213	1.00	
DTi-RSBI	0.133	-0.441*	0.187	0.148	0.047	1.00
	Age	BMI	CCI	SOFA	Intubation period	DTi-RSBI

* The correlation is significant at the 0.05 level.

BMI, body mass index; CCI, Charlson Comorbidity Index; SOFA, sequential organ failure assessment; D-RSBI, respiratory rate divided by half the sum of the right and left diaphragmatic displacements; DTi-RSBI, respiratory rate divided by the product of the diaphragmatic displacement and diaphragm inspiratory time.

Supplementary Table S3. Univariable and multivariable analyses of hospital length of stay ≥ 14 days after ICU discharge

	**Univariable analysis**		**Multivariable analysis (Model 1) [a]**		**Multivariable analysis (Model 2) [a]**		**Multivariable analysis (Model 3) [b]**		**Multivariable analysis (Model 4) [b]**	
	**OR (95% CI)**	***P*-value**	**aOR (95% CI)**	***P*-value**	**aOR (95% CI)**	***P*-value**	**aOR (95% CI)**	***P*-value**	**aOR (95% CI)**	***P*-value**
**Age**	1.02 (0.96–1.08)	0.552	1.01 (0.95–1.07)	0.835	1.00 (0.94–1.07)	0.992	1.01 (0.92–1.11)	0.813	1.00 (0.91–1.10)	0.990
**Male**	0.94 (0.19–4.70)	0.944	0.85 (0.14–5.09)	0.858	0.77 (0.12–4.76)	0.778	1.25 (0.03–52.25)	0.908	0.32 (0.01–22.19)	0.324
**BMI (kg/m²)**	0.94 (0.82–1.06)	0.314	0.99 (0.86–1.14)	0.890	1.00 (0.87–1.16)	0.991	0.97 (0.78–1.22)	0.815	1.04 (0.81–1.33)	0.762
**Smoking**	0.46 (0.11–1.90)	0.283					0.25 (0.01–7.06)	0.253	0.41 (0.02–10.24)	0.585
**CCI**	1.06 (0.80–1.39)	0.699					1.03 (0.64–1.66)	0.898	0.89 (0.53–1.47)	0.639
**SOFA at extubation**	1.25 (0.90–1.72)	0.178					1.27 (0.77–2.12)	0.353	1.10 (0.66–1.84)	0.723
**EF <50%**	1.93 (0.41–9.10)	0.407					0.37 (0.02–8.33)	0.533	0.31 (0.01–9.17)	0.497
**Duration of mechanical ventilation (day)**	1.54 (1.12–2.12)	0.007					1.60 (1.09–2.35)	0.016	1.93 (1.13–3.30)	0.016
**D-RSBI**	11.17 (1.10–113.12)	0.041	10.22 (0.87–120.78)	0.065			19.65 (0.45–855.51)	0.122		
**DTi-RSBI**	14.96 (1.42–157.93)	0.024			15.80 (1.07–233.35)	0.045			496.96 (1.28–192949.872)	0.041

[a] Models 1 and 2 were adjusted for age, sex, and BMI.

[b] Models 3 and 4 were adjusted for all variables.

OR, odds ratio; aOR, adjusted odds ratio; CI, confidence interval; BMI, body mass index; CCI, Charlson Comorbidity Index; SOFA, sequential organ failure assessment; EF, ejection fraction; RSBI, rapid shallow breathing index; DD, diaphragmatic displacement; DTi, product of diaphragmatic displacement and diaphragm inspiratory time; D-RSBI, respiratory rate divided by half the sum of the right and left diaphragmatic displacements; DTi-RSBI, respiratory rate divided by the product of diaphragmatic displacement and diaphragm inspiratory time

Supplementary Table S4. Univariable and multivariable analyses of mortality after ICU discharge

	**Univariable analysis**		**Multivariable analysis (Model 1)** [a]		**Multivariable analysis (Model 2)** [a]		**Multivariable analysis (Model 3)** [b]		**Multivariable analysis (Model 4)** [b]	
	**OR (95% CI)**	***P*-value**	**aOR (95% CI)**	***P*-value**	**aOR (95% CI)**	***P*-value**	**aOR (95% CI)**	***P*-value**	**aOR (95% CI)**	***P*-value**
**Age**	1.00 (0.94–1.07)	0.928	1.00 (0.93–1.07)	0.992	1.00 (0.93–1.07)	0.903	0.94 (0.83–1.07)	0.350	0.94 (0.82–1.07)	0.337
**Male**	0.48 (0.09–2.59)	0.391	0.31 (0.04–2.15)	0.235	0.29 (0.04–2.09)	0.222	0.01 (0.00–2.06)	0.086	0.01 (0.00–2.21)	0.090
**BMI (kg/m^2)**	0.80 (0.65–1.00)	0.046	0.81 (0.63–1.03)	0.085	0.81 (0.63–1.03)	0.086	0.68 (0.41–1.15)	0.148	0.68 (0.40–1.15)	0.151
**Smoking**	1.25 (0.26–6.07)	0.782					29.32 (0.30–2837.29)	0.148	24.32 (0.28–2147.38)	0.163
**CCI**	1.01 (0.74–1.38)	0.939					1.15 (0.63–2.10)	0.640	1.12 (0.61–2.04)	0.723
**SOFA at extubation**	1.24 (0.87–1.77)	0.236					1.11 (0.70–1.76)	0.657	1.05 (0.67–1.66)	0.829
**EF <50%**	4.76 (0.90–25.30)	0.459					8.94 (0.44–183.12)	0.155	10.35 (0.50–213.40)	0.130
**Duration of mechanical ventilation (day)**	1.04 (0.83–1.31)	0.728					1.15 (0.78–1.69)	0.477	1.18 (0.80–1.74)	0.411
**D-RSBI**	3.84 (0.75–19.58)	0.105	2.19 (0.35–13.63)	0.402			2.42 (0.28–21.26)	0.424		
**DTi-RSBI**	5.89 (0.70–49.28)	0.102			2.99 (0.28–31.87)	0.365			2.87 (0.21–38.80)	0.427

[a] Models 1 and 2 were adjusted for age, sex, and BMI.

[b] Models 3 and 4 were adjusted for all variables.

OR, odds ratio; aOR, adjusted odds ratio; CI, confidence interval; BMI, body mass index; CCI, Charlson Comorbidity Index; SOFA, sequential organ failure assessment; EF, ejection fraction; RSBI, rapid shallow breathing index; DD, diaphragmatic displacement; DTi, product of diaphragmatic displacement and diaphragm inspiratory time; D-RSBI, respiratory rate divided by half the sum of the right and left diaphragmatic displacements; DTi-RSBI, respiratory rate divided by the product of diaphragmatic displacement and diaphragm inspiratory time
